# Brain regionalization genes are co-opted into shell field patterning in Mollusca

**DOI:** 10.1038/s41598-017-05605-5

**Published:** 2017-07-14

**Authors:** Tim Wollesen, Maik Scherholz, Sonia Victoria Rodríguez Monje, Emanuel Redl, Christiane Todt, Andreas Wanninger

**Affiliations:** 1 Department of Integrative Zoology, Faculty of Life Sciences, University of Vienna, Althanstraße 14, 1090 Vienna, Austria; 2 University Museum of Bergen, University of Bergen, Allégaten 41, 5007 Bergen, Norway

## Abstract

The ‘brain regionalization genes’ *Six3/6*, *Otx*, *Pax2/5/8*, *Gbx*, and *Hox1* are expressed in a similar fashion in the deuterostome, ecdysozoan, and the cephalopod brain, questioning whether this holds also true for the remaining Mollusca. We investigated developmental *Gbx*-expression in representatives of both molluscan sister groups, the Aculifera and Conchifera. *Gbx* is expressed in the posterior central nervous system of an aculiferan polyplacophoran and solenogaster but not in a conchiferan bivalve suggesting that *Gbx*, together with *Six3/6*, *Otx*, *Pax2/5/8*, and *Hox1*, is involved in central nervous system regionalization as reported for other bilaterians. *Gbx* is, however, also expressed in the anterior central nervous system, i.e. the anlagen of the cerebral ganglia, in the solenogaster, a condition not reported for any other bilaterian so far. Strikingly, all *Gbx-*orthologs and the other ‘posterior brain regionalization genes’ such as *Pax2/5/8* and *Hox1* are expressed in the mantle that secretes shell(s) and spicules of mollusks (except cephalopods). In bivalves, the ancestral condition has even been lost, with *Gbx* and *Pax2/5/8* not being expressed in the developing central nervous system anymore. This suggests an additional role in the formation of the molluscan shell field(s) and spicule-bearing cells, key features of mollusks.

## Introduction

Mollusca is one of the largest bilaterian clades and accounts for an incredible diversity of body plans. Recent phylogenomic studies have supported a sister group relationship of the Aculifera and the Conchifera^1–3^. Aculiferans include the worm-shaped aplacophoran Solenogastres (Neomeniomorpha) and Caudofoveata (Chaetodermomorpha). These clades are characterized by a mantle covered with cuticle and mineralized sclerites (spicules) instead of a shell or shell plates. Polyplacophora (chitons) constitutes the third aculiferan clade and exhibits eight shell plates surrounded by cuticle and, in some cases, scales and/or spicules. All remaining molluscan class-level taxa, such as Scaphopoda, Bivalvia, Gastropoda, Monoplacophora, and Cephalopoda, belong to the Conchifera having a single shell, except for bivalves with two shell valves. While adult bivalves and scaphopods are benthic animals, many with a pronounced foot, the majority of cephalopods has internalized their shell in adaption to a life style as motile predators. Among conchiferans the phylogenetic interrelationships are far from being settled, rendering it difficult to infer how body plans have evolved^4^.

The molluscan central nervous system (CNS) is composed of two pairs of longitudinal nerve cords (tetraneurous) and it is centralized to different degrees. Among the Aculifera, polyplacophorans have rather few ganglia, while solenogastres and caudofoveates exhibit more ganglia in particular in the anterior body region^5^. Except for monoplacophorans that possess a CNS with few ganglia, the conchiferan CNS is ganglionated to a higher degree, most apparent in the compact cephalopod brain^6–8^. In bilaterian model organisms such as fruit fly and mouse certain homeobox genes are expressed in a staggered fashion in the CNS during ontogenesis^9, 10^. These genes are involved in the regionalization of the developing CNS into individual domains such as the vertebrate midbrain-hindbrain boundary^9^. *Six3/6* and *Otx* are expressed in the anteriormost CNS regions, followed by an intermediate *Pax2/5/8-*expression domain and a posterior *Gbx*-expression domain with co-expression of anterior Hox genes^9^. Recent studies unraveled that this gene expression profile is also present in cephalopods^11–15^. Data on other mollusks, in particular with respect to the expression of the ANTP-class gene *Gbx/unplugged* (*gastrulation brain homeobox*), are scarce, but of importance to understand the evolution and development of the molluscan CNS.

In order to infer whether *Gbx* is also expressed in the CNS of non-cephalopod mollusks, we investigated its expression during ontogenesis in the aculiferans *Acanthochitona crinita* (a polyplacophoran) and *Wirenia argentea* (an aplacophoran solenogaster), as well as in the conchiferan protobranch bivalve *Nucula tumidula*.

## Methods

### Collection and culture of animals

Adults of the polyplacophoran *Acanthochitona crinita* (Pennant, 1777) were collected in summer 2012 in the intertidal zone close by the Station Biologique Roscoff (Roscoff, France). Animals were spawned and developmental stages were reared at 20 °C as described previously^15^. Adults of the aplacophoran *Wirenia argentea* Odhner, 1921 and the bivalve *Nucula tumidula* Malm, 1861 were collected at depths of 190–226 m and 227–312 m with a hyperbenthic sled on the silty seafloor in Hauglandsosen and Hjeltefjorden, respectively (Bergen, Norway) in March 2012, from November 2012 to January 2013, and from November to December 2013. Animals were spawned and developmental stages were reared at 7 °C in the dark as described previously^15, 16^.

### RNA extraction and fixation of animals for *in situ* hybridization experiments

For all three species, several hundred individuals of different developmental stages were investigated. For the polyplacophoran *Acanthochitona crinita* and the bivalve *Nucula tumidula* developmental stages ranging from gastrulae to postmetamorphic individuals were collected. For the solenogaster *Wirenia argentea* adults as well as freshly hatched test-cell larvae (0–1 days after hatching (dph), early test-cell larvae (6–7 dph), mid-stage test-cell larvae (10–11 dph), and late test-cell larvae (14–15 dph)) were collected. The above-mentioned larvae were fixed for *in situ* hybridization experiments and used for RNA extraction as previously described^15–17^.

### RNAseq and transcriptome assembly

Total RNA from pooled developmental stages of *Acanthochitona crinita* and *Nucula tumidula*, respectively, and pooled RNA from developmental stages and adults of *Wirenia argentea* was sequenced by Illumina technology (Eurofins, Ebersberg, Germany). Paired-end reads of an average read length of 100 bp were obtained and were subsequently filtered (rRNA removal; see refs 15 and 16 for details on transcriptome assembly). Adapter and low quality sequences were trimmed, normalized, and assembled *de novo* into contigs with the assembler Trinity^18^ or IDBA-tran, Version 1.1.1 in case of *W*. *argentea* ^16, 19^.

### Alignment and phylogenetic analysis

Homeodomain amino acid sequences of Gbx, Dlx, Hox1, Hox3, Cdx, and Lhx2/4 were retrieved from the homeobox database (http://homeodb.cbi.pku.edu.cn/) and GenBank (accession numbers provided in Supplementary Fig. 1)^20, 21^. Gbx amino acid sequences were used in BLAST searches against the assembled transcriptomes of *Acanthochitona crinita*, *Wirenia argentea*, and *Nucula tumidula*. Predicted amino acid sequences of Acr-Gbx, War-Gbx, and Ntu-Gbx and the above-mentioned homeodomains of other homeobox sequences were aligned using MAFFT v7.123b^22^. The alignment was edited manually with Aliview v1.18 and used for the phylogenetic analysis. The phylogenetic analysis presented in this study includes the predicted amino acid sequences of cloned *Gbx* orthologs of *A*. *crinita*, *W*. *argentea*, and *N*. *tumidula*. The bayesian phylogenetic analysis was carried out with MrBayes v3.2.5 with LG model of amino acid replacement^23^ estimated with Prottest3 v3.4.2.^24^, gamma-distributed rates, 22.500.000 generations and sampling frequency of 1.000. The phylogenetic tree was manually rooted using FigTree v1.4.1.^25^.

### Molecular isolation of RNA transcripts

First-strand cDNA synthesis of the RNA pooled from different developmental stages of *Acanthochitona crinita*, *Wirenia argentea*, *and Nucula tumidula*, respectively, was carried out by reverse transcription using the First strand cDNA Synthesis Kit for rt-PCR (Roche Diagnostics GmbH, Mannheim, Germany; see also^15, 16^ for the experimental procedure). Identified *Gbx* orthologs were used to design gene-specific primers and PCR products were size-fractioned by gel electrophoresis, gel bands of the expected lengths were excised and cleaned up using a QIAquick Gel Extraction Kit (QIAgen, Hilden, Germany). By insertion into pGEM-T Easy Vectors (Promega, Mannheim, Germany) cleaned-up products were cloned. Plasmid minipreps were grown overnight, cleaned-up with the QIAprep Spin MiniprepKit (QIAgen), and sent off for sequencing. *A*. *crinita-Gbx* (*Acr-Gbx*), *W*. *argentea-Gbx* (*War-Gbx*), and *N*. *tumidula-Gbx* (*Ntu-Gbx*) sequences were identified using the BLASTx algorithm screening the database of the NCBI. All three cloned nucleotide sequences as well as their deduced amino acid sequences were submitted to GenBank (Accession numbers: *Acr-Gbx*: KY500990, *War-Gbx*: KY500991, *Ntu-Gbx*: KY500992).

### Probe synthesis and whole-mount *in situ* hybridization

**Figure 1:**
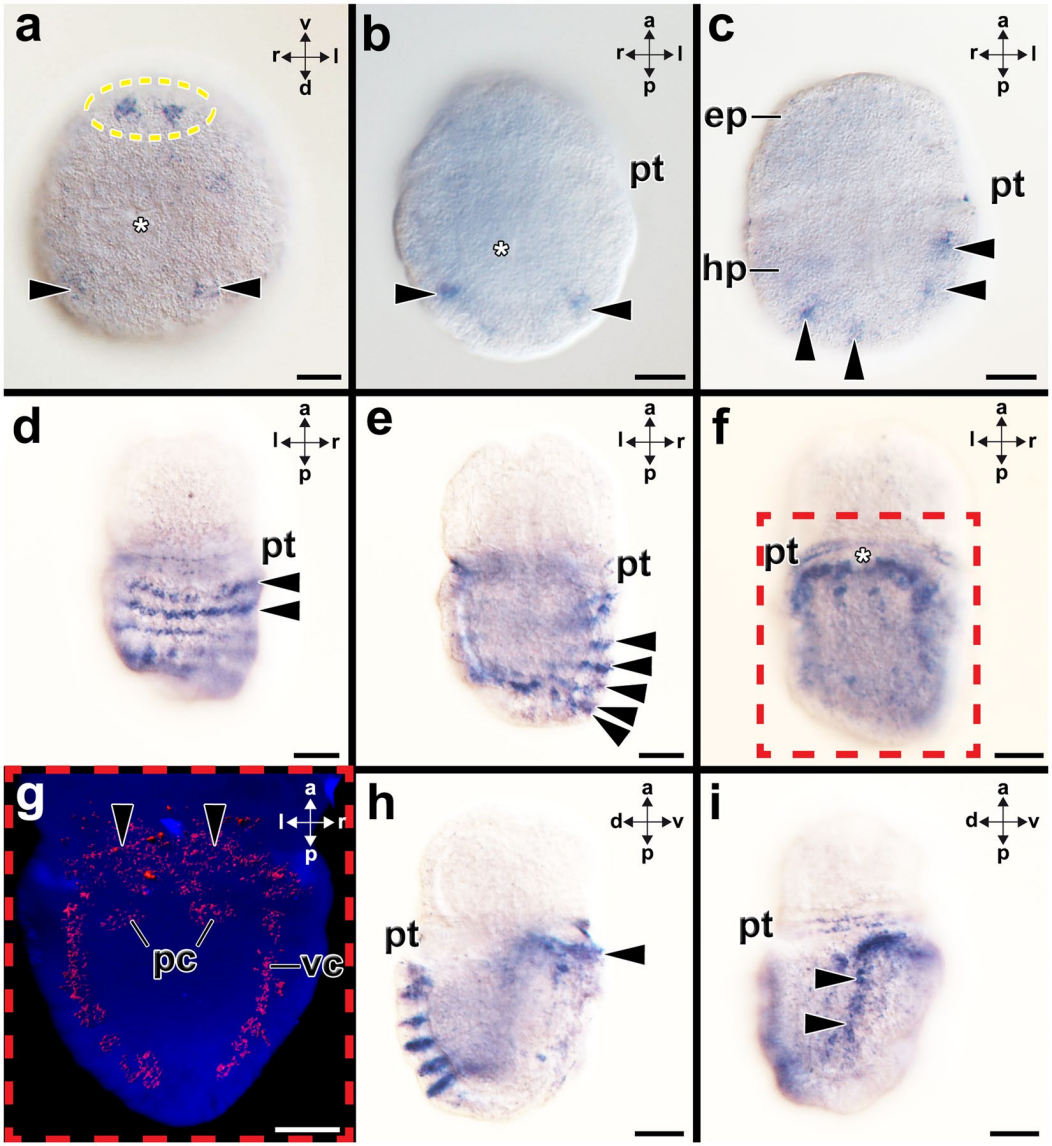
Expression of *Gbx* during embryonic and early larval development of the polyplacophoran *Acanthochitona crinita*. Dorsal (d)-ventral (v), anterior (a)-posterior (p), and left (l)-right (r) axes indicate the orientation. Asterisks mark the blastopore/mouth opening. (**a**) Late gastrulae (4 hpf; ventro-posterior view) expresses *Gbx* in cells in the ventral (encircled) and dorsal regions (arrowheads). (**b-c**) In further developed early trochophore larvae (12 hpf) *Gbx*–expressing cells (arrowheads) are located in the ventral hyposphere (hp). (**d**–**i**) Mid-stage trochophore (35 hpf). (**d**,**e**) This dorsal (**d**) and more ventral optical section (**e**) show *Gbx*-expressing cells (arrowheads) in seven rows of the shell fields. Note the unspecific staining in the prototroch. (**f**,**g**) *Gbx* is also expressed in the ventro-lateral hyposphere in the CNS, an area that is highlighted and 3-D reconstructed in (**g**) (red-dashed box). *Gbx* is expressed in the anterior pedal nerve cord (pc) and the entire visceral nerve cord (vc). *Gbx* is expressed in the region of the pedal nerve cord commissure (arrowheads). (**h**) Note *Gbx*-expression in the dorsal shell fields and the nervous system of the foot (arrowhead). (**i**) *Gbx*–expression in the visceral nerve cord (arrowheads). Abbreviations: ep, episphere; pt, prototroch. Scale bars: 20 µm.

**Figure 2:**
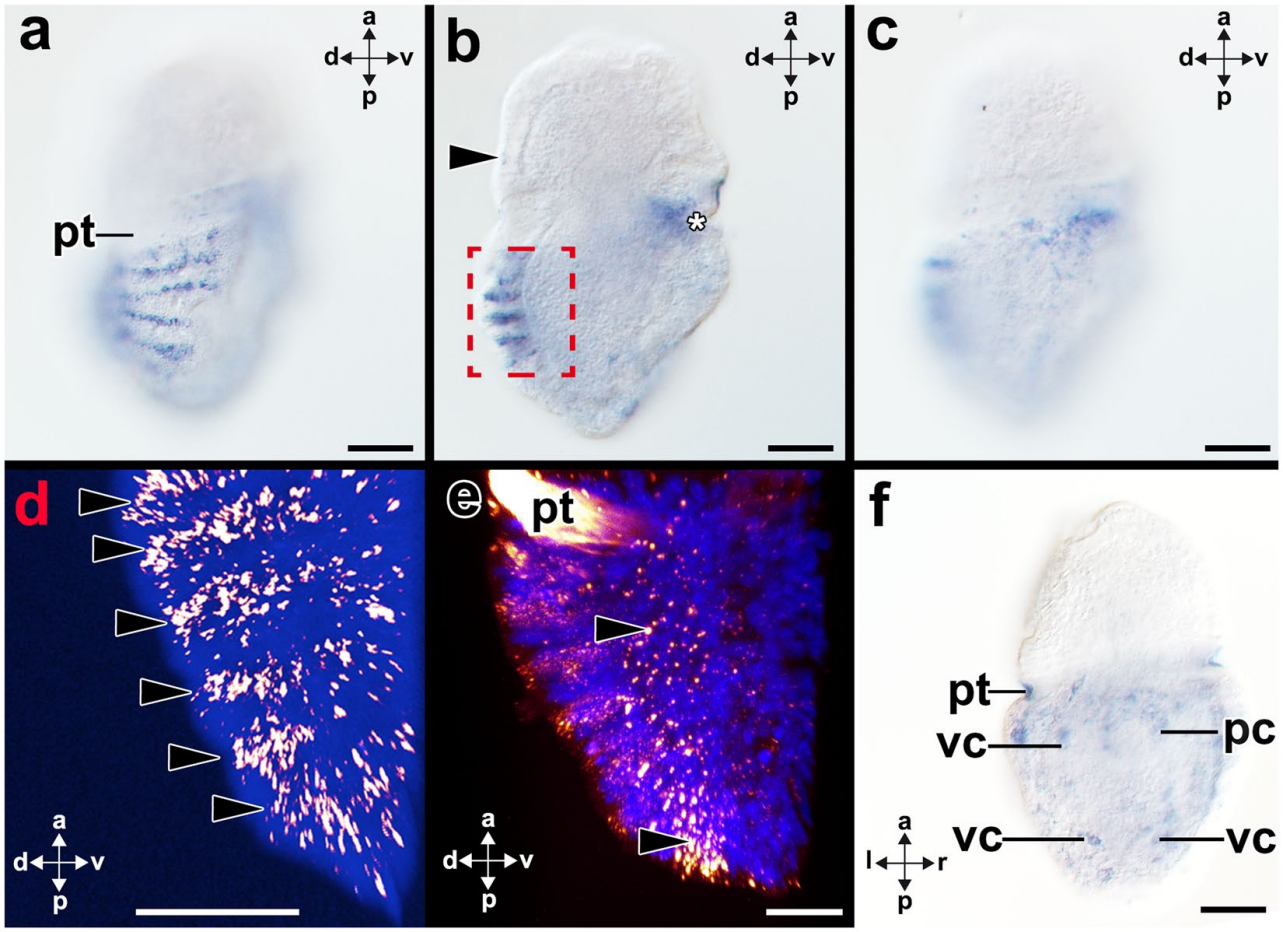
Optical section series of *Gbx*-expression in the metamorphic competent trochophore larva (65 hpf) of the polyplacophoran *Acanthochitona crinita*. Dorsal (d)-ventral (v), anterior (a)-posterior (p), and left (l)-right (r) axes indicate the orientation. Anterior faces up in all micrographs. (**a**–**c**) Sequence of three consecutive optical sections through a metamorphic competent trochophore larva. *Gbx* is expressed in cells of the shell fields, close to the mouth (asterisk) in the area of the pedal commissure, and in the spicule-bearing cells of the perinotum (arrowhead). *Gbx-*expressing cells are located in the outermost cell layer of the mantle that constitutes the shell fields. A reflection scan of the boxed region shown in (**b**) is highlighted in (**d**). (**d**) Reflection scan showing *Gbx-*expression in six rows of cells (arrowheads) in the shell fields (Dapi nuclear counterstain in blue). The seventh row is not included in this micrograph. (**e**) Fluorescent *in situ* hybridization (FISH) staining showing *Gbx*–expression in the spicule-bearing cells (arrowheads) in the area of the prospective perinotum (DAPI nuclear counterstain in blue). Staining in the prototroch (pt) is unspecific. (**f**) Cells of the pedal (pc) and visceral nerve cords (vc) express *Gbx*. Scale bars: 20 µm.

**Figure 3:**
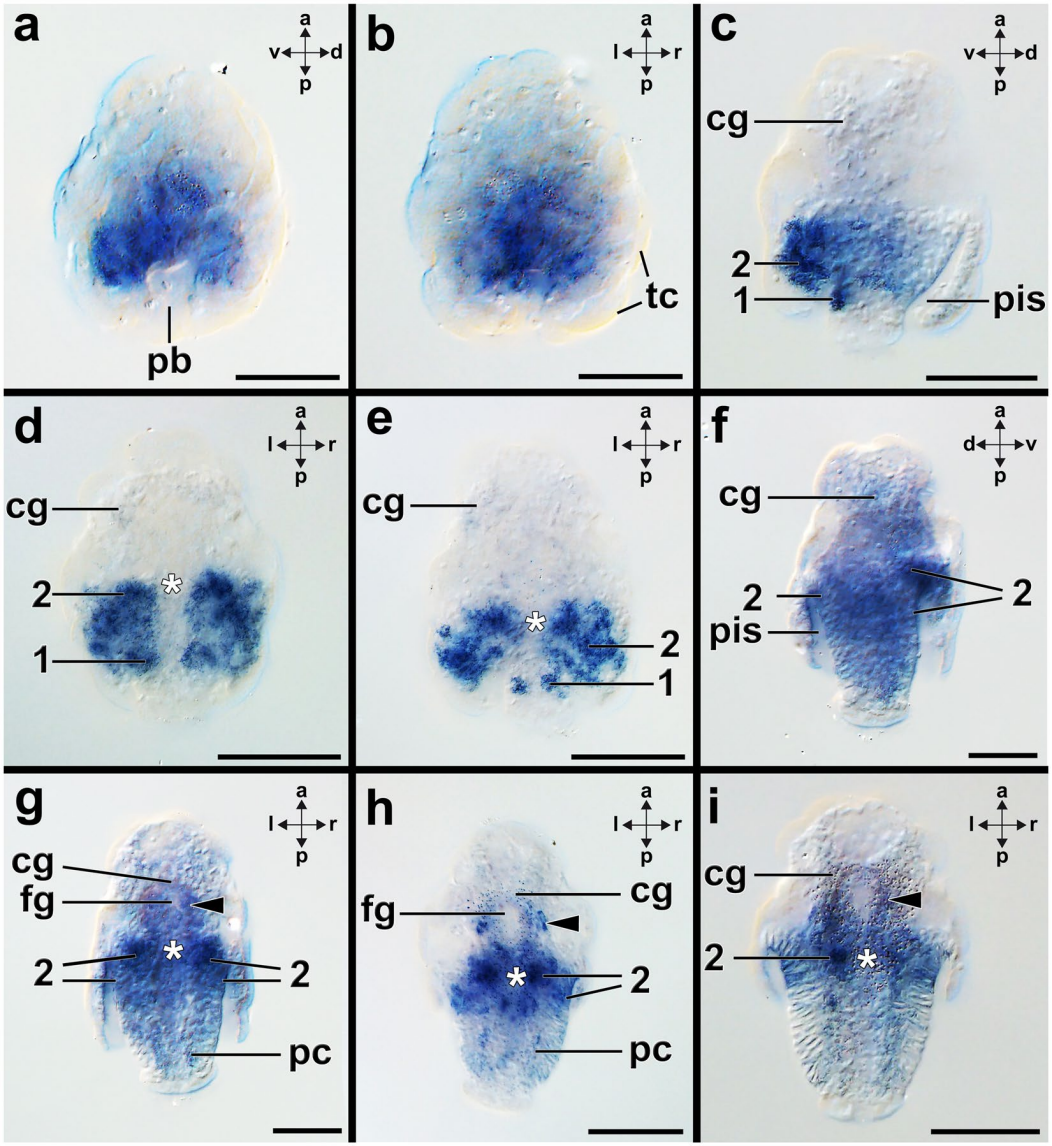
Expression of *Gbx* during ontogenesis of *Wirenia argentea*. Anterior (a)-posterior (p), dorsal (d)-ventral (v), and left (l)-right (r) axes indicate the orientation. Anterior faces up in all micrographs. Asterisks mark the mouth opening. (**a,b**) Freshly hatched larvae (0–1 dph) express *Gbx* in a dorso-ventral gradient with the highest expression in the ventral epidermal cells close to the pseudo-blastopore (pb). The early embryo possesses flattened and large test-cells (tc) that do not express *Gbx*. (**c**–**e**) Early larvae (6–7 dph) express *Gbx* ventral laterally in epidermal cells (2) that line the peri-imaginal space (pis). This expression domain extends towards the lateral portions of the mouth and also includes subepidermal cells (2). Two bilateral groups of *Gbx*-expressing cells (1) are located ventrally at the posterior pole of the outgrowing trunk. These expression domains might be associated with the developing pedal nerve cords. Note that the trunk is less retracted in (**e**) compared to (**d**) and therefore *Gbx*-expression domain “1” is clearly distinguishable from other expression domains. The developing cerebral ganglia (cg) show faint *Gbx*-expression. (**f**–**g**) Mid-stage larvae (10–11 dph) express *Gbx* in the developing pedal nerve cords. *Gbx* is still expressed in epidermal cells (2) which line the peri-imaginal space and in subepidermal cells close to the latter cells (2). Some individuals show *Gbx*-expression adjacent to the developing foregut (fg) (arrowheads). *Gbx* is also expressed in the region of the developing cerebral ganglia. (**h**,**i**) Late larvae (14–15 dph) express *Gbx* in the developing pedal nerve cords. *Gbx* is expressed ventro-laterally in epidermal cells (2) adjacent to the peri-imaginal space and in subepidermal cells close to the latter cells (2). This expression domain extends towards the lateral portions of the mouth. *Gbx*-expression is also visible adjacent to the developing foregut (arrowheads). Abbreviations: pc, pedal nerve cords. Scale bars: 50 µm.

**Figure 4:**
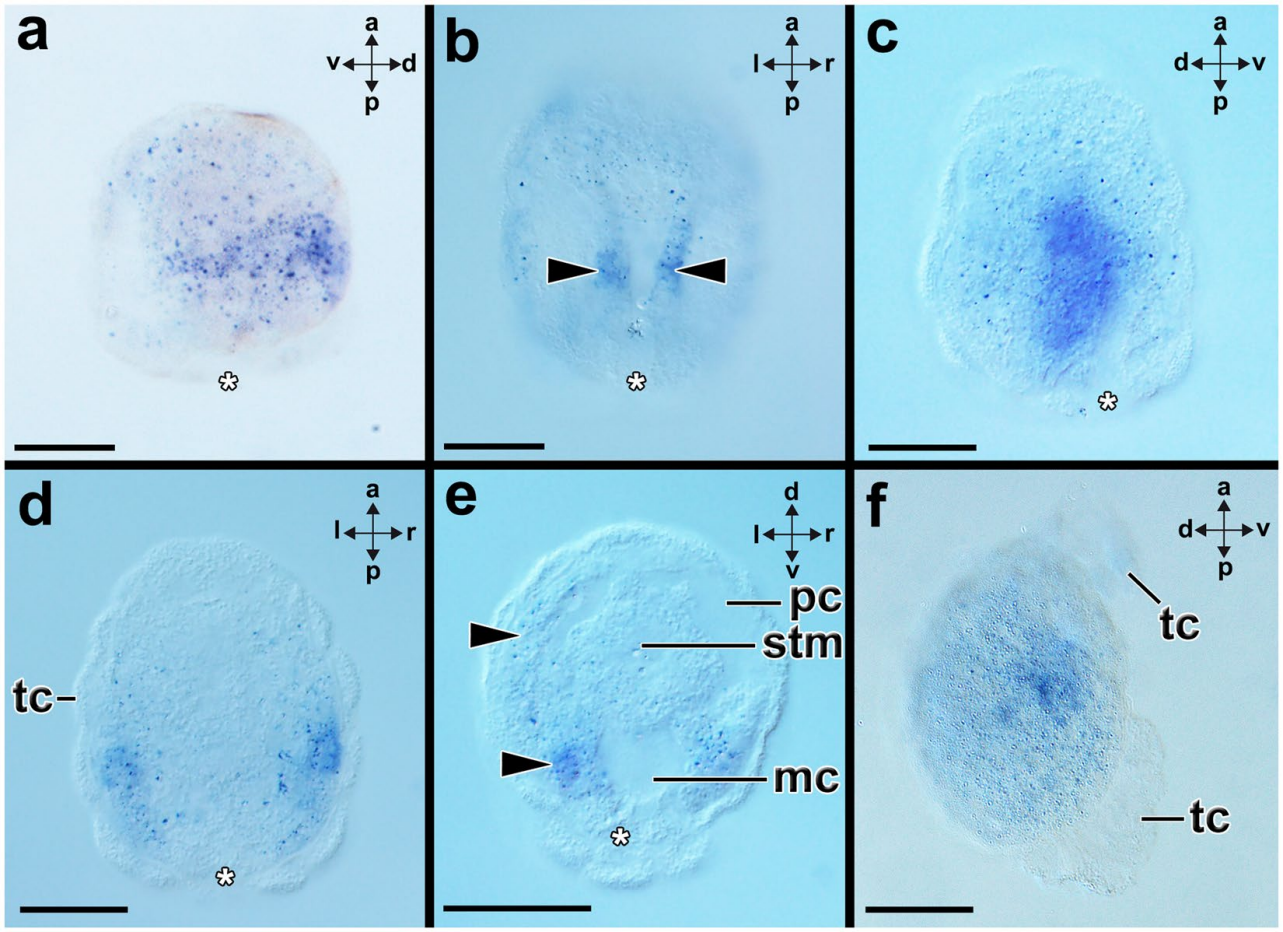
Expression of *Gbx* during ontogenesis of the bivalve *Nucula tumidula*. Anterior (a)-posterior (p), dorsal (d)-ventral (v) and left (l)-right (r) axes indicate the orientation. The blastopore (asterisk) leads to the definitive mouth. (**a**) In early test-cell larva (3 dpf) *Gbx* is expressed in the ectoderm on the dorsal and lateral sides. (**b**) In 8 dpf old test-cell larvae *Gbx* is expressed in the ventral mantle margins (arrowheads). (**c**,**d**) In further developed specimens (12 dpf) *Gbx* is expressed laterally in the mantle. The large and flattened ciliated test-cells (tc) constitute the outer cellular layer of the larva and do not express *Gbx*. (**e**) Late test-cell larvae (12 dpf) express *Gbx* in the region of the ventral (lower arrowhead) and lateral mantle (upper arrowhead). Note the mantle cavity (mc), the perivisceral cavity (pc), and the stomache (stm). (**f**) Settled, early postmetamorphic individuals (22 hpf) express *Gbx* in parts of the mantle close to the foot. The test-cells have been shed during metamorphosis and will eventually be ingested. Scale bars: 30 µm (except Change “I” into “f”: 50 µm).

The detailed experimental procedure was described previously^15, 16^. Riboprobe templates were amplified via standard PCR from miniprepped plasmids using M13 forward and reverse primers. *In vitro* transcription reactions were performed with these templates, digoxigenin-UTP (DIG RNA Labeling Kit, Roche Diagnostics), and SP6/T7 polymerase (Roche Diagnostics GmbH) for the syntheses of antisense riboprobes, according to the manufacturer’s instructions. In whole-mount *in situ* hybridization experiments, specimens were rehydrated into PBT (phosphate buffered saline +0.1% Tween-20) and treated with Proteinase-K at 37 °C for 10 min. Developmental stages of *Acanthochitona crinita* were Proteinase-K treated with 60 µg/ml in PBT and those of *Wirenia argentea* and *Nucula tumidula* with 10 µg/ml in PBT. Specimens were prehybridized in hybridization buffer for 4 h either at 65 °C (*A*. *crinita*) or at 56 °C (*W*. *argentea*, *N*. *tumidula*). Hybridization was performed at the same temperature with probe concentrations ranging between 0.5 and 1 μg/ml for 21–24 h. A DIG-labeled AP-antibody was used at a dilution of 1:5000 in blocking solution at 4 °C over night. Color development in the NBT/BCIP/Alkaline Phosphatase buffer solution took 6–24 hrs at 4 °C. For *A*. *crinita* and *N*. *tumidula*, a minimum of 40 individuals per stage was investigated, while a minimum of 20 individuals per stage were used for *W*. *argentea*. Fluorescent *in situ* hybridization was performed with Fast Blue (Sigma-Aldrich) on *A*. *crinita* as described previously^27, 28^. The majority of whole-mount preparations was cleared in a solution of benzyl-benzoate and benzyl alcohol, mounted on objective slides, and analyzed. After *in situ* hybridization, some specimens of *A*. *crinita* were embedded in O. C. T. medium (VWR, Vienna, Austria) and cut into 15–30 μm cryosections with a cryotome (Leica CM 3050S). For counterstains of cell nuclei, sections were stained with DAPI (Sigma- Aldrich, St. Louis, MO, USA), washed in phosphate buffered saline and subsequently mounted in Fluoromount G (Southern Biotech, Birmingham, Alabama, USA). Preparations were documented with an Olympus BX53 Microscope (Olympus, Hamburg, Germany). In addition, developmental stages were scanned with a Leica confocal SP5 II microscope (Leica Microsystems, Wetzlar, Germany) using bright-field, autofluorescence, and reflection mode scans to understand the precise location of transcripts^29^. If necessary, images were processed with Adobe Photoshop 9.0.2 software (Adobe Systems, Inc; San Jose, CA, USA) to adjust for contrast and brightness (Figs 1, 2, 3 and 4). Sketch drawings were created with Adobe Illustrator CC 2015.1.0 (Adobe Systems, Inc). Fluorescence labeling of filamentous F-actin with Alexa Fluor 488 was performed on 7 dph old larvae of *W*. *argentea* as described previously^26^.

## Results

### *Gbx* gene orthologs and phylogenetic analysis

Acr-Gbx, War-Gbx, and Ntu-Gbx show high sequence similarity with their bilaterian orthologs as revealed by multiple amino acid sequence alignment. The phylogenetic analysis shows that all sequences cluster with their bilaterian orthologs with high support (Supplementary Figs 1 and 2).

### *Gbx*-expression in the polyplacophoran *Acanthochitona crinita*

**Figure 5:**
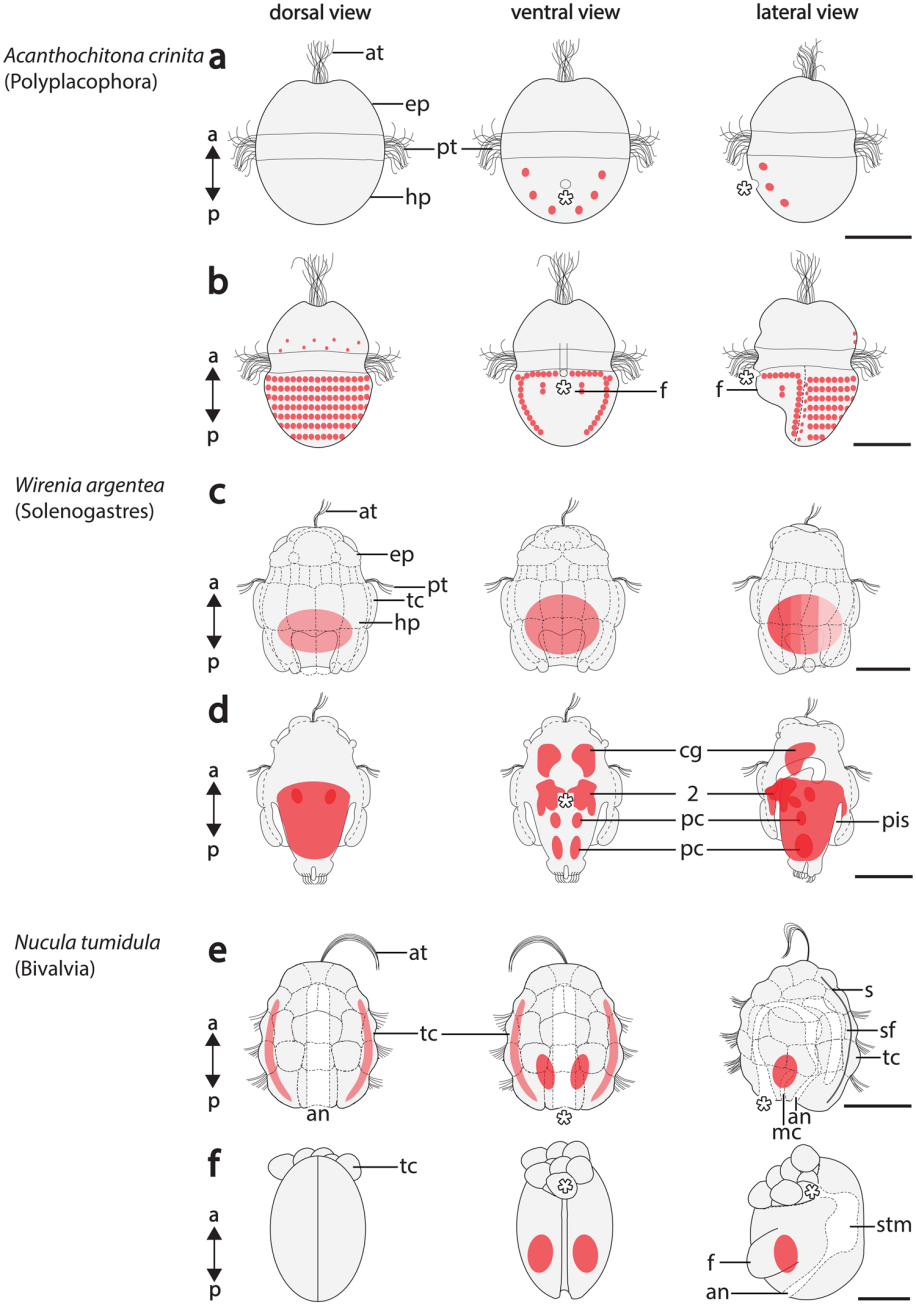
Summary of *Gbx*-expression (red) during polyplacophoran, solenogaster, and bivalve development. Anterior (a)-posterior (p) axes indicate the orientation. Asterisks mark the mouth opening. (**a**) Early trochophore larva (12 hpf) of the polyplacophoran *Acanthochitona crinita* possess an episphere (ep) that is divided from the hyposphere (hp) by a prototroch (pt). The anteriormost region of the episphere exhibits an apical organ with a (ciliary) apical tuft (at). (**b**) Metamorphic competent individual (65 hpf) of *A*. *crinita* showing seven shell fields with each one row of *Gbx*-expressing cells on the dorsal side and a foot (f) on the ventral side. (**c**) Early test-cell larva (0–1 dph) of the solenogaster *Wirenia argentea* exhibit large and flattened test-cells (tc). (**d**) In mid-stage test-cell larva (10–11 dph) of the solenogaster *W*. *argentea Gbx* is expressed in the anlagen of the cerebral ganglia (cg), in cells (2) that line the peri-imaginal space (pis) and in cells that are associated with the pedal nerve cords (pc). (**e**) Late test-cell larva (12 dpf) of the bivalve *Nucula tumidula* possess large and flattened test-cells, a mouth, and anus (an). The shell fields (sf) give rise to both shell valves (s). (**f**) Postmetamorphic individuals (22 dpf) of *N*. *tumidula* shed all test-cells which are subsequently ingested. Abbreviations: mc, mantle cavity; stm, stomach. Scale bars: 50 µm.

*Gbx* is expressed in cells that are located in ventral and dorsal regions of late gastrulae (Fig. 1a). Gastrulae subsequently develop into early trochophore larvae that are characterized by a ciliary band, the prototroch, that divides the anterior episphere from the posterior hyposphere (Figs 1b,c and 5a). Trochophore larvae possess an apical organ with a ciliary tuft in the anterior episphere, a mouth in the anterior ventral hyposphere and seven shell fields that develop on the dorsal hyposphere (Fig. 5a). In early trochophore larvae *Gbx*–expressing cells are only located in the ventral hyposphere (Figs 1b,c and 5a). Mid-stage trochophore larvae express *Gbx* in cells of the seven shell fields (Fig. 1d,e,h). Each shell field is ridge-like and all *Gbx-*expressing cells are located in the epidermis (Supplementary Fig. 3a,c). *Gbx*-transcripts are also present in the distal-most portion of spicule-bearing cells of the dorsal episphere (red-lined arrowhead in Supplementary Fig. 3a). In addition, *Gbx*-expressing cells are associated with the visceral and pedal nerve cords (Fig. 1f–i; Supplementary Fig. 3c–f). While cells along the entire length of the visceral nerve cords express *Gbx*, only cells along the anteriormost portion of the pedal nerve cord express *Gbx* (Fig. 1f,g,i). In the anteriormost portion of the hyposphere, *Gbx* is expressed in the pedal commissures (arrowheads in Fig. 1g). In metamorphic competent trochophore larvae, *Gbx-*expressing cells are located in seven rows in the prospective shell fields of the hyposphere as seen in mid-stage trochophores (Figs 2a,b,d and 5b; c.f. Supplementary Fig. 3a). *Gbx*-expressing cells are slender and located in the anterior portion of each shell field (Fig. 2d,e). Visceral and pedal nerve cords exhibit *Gbx-*expressing cells, however, expression is less prominent compared to earlier developmental stages. In contrast to the visceral nerve cords, the pedal nerve cords only exhibit *Gbx-*expressing cells in the anteriormost region as seen in mid-stage trochophores (Figs 2f and 5b; c.f. Supplementary Fig. 3c). Additional *Gbx-*expressing cells are located in the anteriormost portion of the hyposphere, in the region of the pedal commissures (Figs 2b,c and 5b). Spicule-bearing cells in the prospective perinotum, i.e. the areas surrounding the shell fields, also express *Gbx* (Fig. 2b,e). In some animals unspecific staining was observed attached to the trochoblasts (Fig. 2e).

### *Gbx-*expression in the aplacophoran *Wirenia argentea*

Gastrulae develop into early test-cell larvae that are characterized by large and flattenened test-cells (Fig. 5c ^30^). The apical organ is associated with a tuft and the prototroch divides episphere and hyposphere (Fig. 5c). In freshly hatched larvae, *Gbx* is expressed in a dorsal-ventral gradient with the highest expression in the ventral epidermal cells close to the pseudo-blastopore, a posterior invagination of the larval body (Figs 3a,b and 5c). The pseudo-blastopore does not develop into the actual mouth, instead the larval body (trunk) growths out through it. *Gbx*-expression decreases in the dorso-lateral epidermal cells surrounding the pseudo-blastopore (Fig. 3a,b). Early larvae express *Gbx* latero-ventrally in epidermal cells that line the peri-imaginal space (“2” in Fig. 3c–e). The majority of these cells develop into spicule-bearing cells of the prospective trunk (Supplementary Fig. 4). The latter *Gbx*–expression domain extends laterally towards the mouth and includes also subepidermal cells (“2” in Fig. 3d). On each side two ventrally located spots at the posterior pole of the outgrowing trunk express *Gbx* (“1” in Fig. 3c–e). The developing cerebral ganglia show faint *Gbx*-expression (Fig. 3c,d). Mid-stage larvae express *Gbx* in the developing pedal nerve cords (Figs 3g and 5d), a *Gbx*-expression domain that might correspond to expression domain “1” of early larvae (Fig. 3e). *Gbx* is still expressed in epidermal cells that line the peri-imaginal space and in subepidermal cells close to the latter cells (“2” in Figs 3f,g and 5d). The former epidermal cells contribute to the mantle of the outgrowing trunk (cf. ref. 30). Some individuals show *Gbx*-expression adjacent to the developing foregut (arrowhead in Fig. 3g), expression domains that may correspond to the anlagen of the buccal and/or lateral ganglia^30^. In addition, the anlagen of the cerebral ganglia express *Gbx* (Figs 3f,g and 5d). Late larvae express *Gbx* in their developing pedal nerve cords (Fig. 3h). *Gbx* is still expressed latero-ventrally in epidermal cells that line the peri-imaginal space and in subepidermal cells close to the latter cells (Fig. 3h–i). Expression is strongest on both sides of the mouth opening (“2” in Fig. 3h–i) and co-localized with expression of *Pax6*, *Paxβ*, and *engrailed* ^31^ (Scherholz, unpublished data). *Gbx* is also expressed in cells adjacent to the developing foregut, a region where buccal or lateral ganglia may be located (arrowheads in Fig. 3h–i). In the region of the developing cerebral ganglia, faint *Gbx*-expression is present (Fig. 3h,i).

### *Gbx*-expression in developmental stages of the bivalve *Nucula tumidula*

After gastrulation, a test-cell larva hatches that exhibits three rows of ciliated cells and an apical organ with a ciliary tuft (Fig. 5e). These rows of cilia arise from flattened and large test-cells that constitute the outermost cell layer of the larval body (Fig. 5e). The blastopore leads to the definite mouth of the larva and is located on the posterior pole together with the anus (Fig. 5e). In early test-cell larvae, *Gbx* is expressed in the dorsal ectoderm (Fig. 4a). In further developed larvae, *Gbx* is expressed in the ventral mantle margin (Fig. 4b; for details on the anatomy of the bivalve test-cell larva see ref. 32). Subsequently, larvae cease to express *Gbx* in the ventral mantle regions and lateral mantle areas express *Gbx* (Fig. 4c,d). Late test-cell larvae express *Gbx* again in the region of the ventral mantle and faint expression was detected in the lateral mantle areas (Fig. 4e). Early postmetamorphic individuals express *Gbx* in parts of the mantle close to the foot (Figs 4f and 5f).

### Summary of the main *Gbx*-expression domains in all three molluscan species

*Gbx* is expressed in the posterior CNS of polyplacophoran and aplacophoran larvae but not in the CNS of bivalve larvae (Fig. 5b,d–f). The aplacophoran anteriormost CNS, i.e. the anlagen of the cerebral ganglia, also express *Gbx* (Fig. 5d). In polyplacophorans *Gbx* is expressed in spicule-bearing cells and cells of the shell fields (Fig. 5b). Spicule-bearing cells and cells of the mantle express *Gbx* in the aplacophoran and the bivalve, respectively (Fig. 5c–f).

## Discussion

### The molluscan *Gbx* orthologs are expressed in ectodermal domains

Our phylogenetic analysis includes *Gbx*-orthologs of various other bilaterian representatives and corroborates the identity of *Acr-Gbx*, *War-Gbx*, and *Ntu-Gbx* with high support (Supplementary material Figs 1 and 2). Our results on mollusks corroborate the finding of previous studies that *Gbx* is expressed in the ectoderm of bilaterians^14, 33, 34^. In the aculiferans *Acanthochitona crinita* and *Wirenia argentea* as well as in the conchiferan *Nucula tumidula Gbx* is expressed in the nervous system and/or the mantle.

### Do mollusks share similar gene expression profiles in their CNS with other bilaterians?

Genes such as *Six3/6*, *Otx*, *Pax2/5/8*, *Gbx*, and the anterior Hox genes are expressed in a sequential fashion in the developing CNS of phylogenetically distantly related bilaterians^9, 33^. While a couple of ecdysozoans and deuterostomes have been investigated in detail, only few lophotrochozoans have been studied with respect to the expression patterns of the above-mentioned genes^34^. In the aculiferan mollusks *Acanthochitona crinita* and *Wirenia argentea* as well as in the conchiferan cuttlefish *Sepia officinalis*, *Gbx* is expressed in the nervous system more posteriorly to *Otx-* and *Pax2/5/8*- expression domains (this study^14, 15^, Wollesen, unpublished data). The polyplacophoran *A*. *crinita* expresses *Gbx* in the visceral nerve cords and in the anteriormost pedal nerve cords (Fig. 5b). In *S*. *officinalis Gbx* is expressed in the posteriormost brain region, i.e. in the posterior portion of the subesophageal mass (=palliovisceral ganglia) as well as in the stellate ganglia^14^. In *W*. *argentea Gbx* is also expressed in the posterior CNS, i.e. the pedal nerve cords, however also in the anlagen of the cerebral ganglia (Fig. 5d). The latter finding is of surprise and contrasts the situation in other bilaterians with *Gbx*-expression in more posterior regions, partially co-expressed with Hox genes (see above). Since *W*. *argentea* is the only bilaterian investigated so far that shows *Gbx*-expression in a region located thus far anterior, the most parsimonious explanation is that this expression pattern evolved secondarily. This is of interest since a recent study demonstrated that solenogasters exhibit some rather derived character states such as a secondarily simplified body plan^26, 35^.

Polyplacophoran anterior Hox genes are co-expressed with *Gbx* in the posttrochal larval region, a condition that resembles that of other bilaterians including cephalopods (this study^9, 11, 34, 36–38^). Anterior to the *Gbx*-expression domain, a *Pax2/5/8* domain is located in the interbasal lobes of the cephalopod supraesophageal mass^15^. The interbasal lobes lie adjacent to the esophagus that divide the brain into a supraesophageal mass and a subesophageal mass. *W*. *argentea* also shows *Pax2/5/8* expression in its cerebral ganglia and pedal nerve cords^31^. In contrast to cephalopods, solenogasters, and other bilaterians, *Pax2/5/8* is not expressed in the developing CNS of polyplacophorans, bivalves, and gastropods^15, 39^.

Anterior to the *Pax2/5/8* expression domain an *Otx* expression domain is located in cephalopods^12, 15^. In polyplacophoran trochophore larvae, *Otx* is broadly expressed in the episphere where the anlagen of the cerebral commissure are located^28^. *Pax2/5/8* and *Otx* are both expressed in the region of the developing cerebral ganglia in solenogasters^31^ (Redl *et al*., unpublished results). In the gastropod *Crepidula fornicata Otx* is expressed in the anterior CNS of trochophore and veliger larvae^40^, in contrast to trochophore larvae of the patellogastropod *Patella vulgata* that lack *Otx*-expression in this region^41^. As in other bilaterians *Six3/6* is expressed in the anteriormost region of the CNS in gastropods, cephalopods, polyplacophorans, and solenogasters^13, 28, 40^ (Redl *et al*., unpublished results).

In summary, *Six3/6*, *Otx*, and *Gbx* are expressed in the nervous system of the majority of mollusks investigated so far, while *Pax2/5/8* is only expressed in the developing nervous system of solenogasters and cephalopods. *Pax2/5/8* and *Gbx* are not expressed in the developing nervous system of the bivalve *N*. *tumidula* altogether. *Hox1* is expressed in the cephalopod and polyplacophoran CNS.

### *Gbx* and other brain regionalization genes have been co-opted into molluscan shell field development

*Gbx* is expressed in the mantle of the polyplacophoran *Acanthochitona crinita*, the solenogaster *Wirenia argentea*, and the bivalve *Nucula tumidula* (this study: Fig. 5). In contrast, late prehatching embryos of the cephalopod *Sepia officinalis* do not express *Gbx* in the mantle or the shell gland^14^. Seven horizontal rows of *Gbx*-expressing cells are located in the seven shell fields of the *A*. *crinita* (Fig. 5b). Polyplacophoran shell fields are characterized by a ridge-like appearance and a number of different cells give rise to the shell plates of the polyplacophoran *Ischnochiton rissoa* ^42^. “Goblet cells” are large cells that are located in the center of each shell field and anterior to these cells the slender *Gbx*-expressing cells are situated in *A*. *crinita*. Judging by their location and morphology, the *Gbx*-expressing cells may be identified as “type 2” or “type 4” cells, cells that together with the “goblet cells” secrete the shell plates in *I*. *rissoa* ^42^. Early settled polyplacophorans possess seven shell plates, while the eighth shell plate develops several weeks afterwards^43, 44^. In *W*. *argentea Gbx* is strongly expressed in the ventro-lateral mantle region of the outgrowing trunk, i.e. exactly in the same region where spicule-bearing cells are present (Scherholz, unpublished data). In *N*. *tumidula Gbx* is expressed in the ventral mantle regions and subsequently in lateral mantle regions (Fig. 5e,f). The ventral *Gbx*-expressing mantle regions probably contribute to the growth of both shell valves.

Interestingly, recent studies have shown that *Pax2/5/8* is expressed in horizontal rows of cells in the polyplacophoran mantle that gives rise to the shell plates, as well as in the spicule-bearing cells of the aplacophoran *W*. *argentea* ^15, 31^. *Pax2/5/8* is also expressed in the mantle of *N*. *tumidula*, the cephalopod *Idiosepius notoides*, and in the anterior mantle region of the veliger larva of *H*. *asinina* ^15, 39^. The latter expression domain corresponds to the mantle region that secretes the protoconch I, i.e. the first-formed embryonic shell. In addition to *Pax2/5/8* and *Gbx*, *Hox1* is also expressed in the shell fields of gastropods and polyplacophorans^15, 37, 45–47^.

Since *Gbx*, *Pax2/5/8*, and *Hox1* are expressed in mantle domains that secrete the shell(s) of polyplacophorans and conchiferans, the most parsimonious conclusion is that these genes were already expressed in these regions in the last common ancestor of Mollusca. The precise number of shell field(s) in the last common ancestor of Mollusca remains obscure since seven shell fields were probably present at the base of the Aculifera and only one shell was present in the ground pattern of its sister group, the Conchifera^1–3, 26, 35, 47, 48^. Competing evolutionary scenarios suggest that no mineralized shell(s) but spicules were present in the last common ancestor of Mollusca^47–51^. Coleoid cephalopods probably lost *Gbx* and *Hox1* expression in their shell field during evolution^11, 14^. The vast majority of coleoids secrete a slender internalized “shell” (e.g., a non-mineralized gladius or a cuttlebone), an innovation that may be correlated with the loss of *Gbx* and *Hox1* expression in the shell field during coleoid cephalopod evolution. Recent studies on various bivalve and gastropod species demonstrate that their adult mantle secretomes are rather diverse^52–54^. Each species possesses a unique mantle secretome composed of a majority of lineage- or species-specific genes^54^. Although there is indication that mantle secretomes underlie significant changes with respect to their gene composition, this has not been investigated thoroughly throughout the Mollusca^55–57^. Genes that underlie the formation of the shell field(s) and spicule-bearing cells such as *Gbx*, *Pax2/5/8*, and *Hox1* have been investigated to an even lesser degree.

## Conclusions

This study shows that *Gbx* plays a role during the formation of major molluscan traits, the shells and spicules. *Pax2/5/8*, *Gbx*, and *Hox1* are originally known to be involved in the regionalization of the bilaterian nervous system, however, these ‘brain regionalization genes’ were also co-opted into shell and spicule formation. With exception of *Pax2/5/8*, cephalopods do not express any of the above-mentioned genes in the mantle during early ontogenesis, however, all of them in the brain. In contrast, the ancestral condition has been lost with *Gbx* and *Pax2/5/8* expressed only in the mantle but not in the nervous system in the bivalve *N*. *tumidula*. This suggests an additional role of these typical ‘brain regionalization genes’ in the formation of the shell field(s) and spicule-bearing cells. Together with paleontological evidence, this work and future studies of its kind may infer whether the last common molluscan ancestor had a single shell, multiple shell plates, or no shell at all.

## Electronic supplementary material

Supplementary info
