# Supplementary material for: Brain regionalization genes are co-opted into shell field patterning in Mollusca
Source: Sci Rep. 2017 Jul 14;7:5486. doi: 10.1038/s41598-017-05605-5 (PMC5511173; doi:10.1038/s41598-017-05605-5)
Supplement: Supplementary file 1 — Supplementary info [file 41598_2017_5605_MOESM1_ESM.pdf]

Supplementary material:

Brain regionalization genes are co-opted into shell field patterning in Mollusca

Tim Wollesen[a*], Maik Scherholz[a], Sonia Victoria Rodríguez Monje[a], Emanuel Redl[a], Christiane Todt[b], and Andreas Wanninger[a]

[a] Department of Integrative Zoology, Faculty of Life Sciences, University of Vienna, Althanstraße 14, 1090 Vienna, Austria.

[b] University Museum of Bergen, University of Bergen, Allégaten 41, 5007 Bergen, Norway.

Email: Tim Wollesen: tim.wollesen@univie.ac.at

Orcid ID: 0000-0003-0464-1254

Maik Scherholz: maik.scherholz@univie.ac.at

Sonia Victoria Rodríguez Monje: sonia.rodriguez@univie.ac.at

Emanuel Redl: emanuel.redl@univie.ac.at

Andreas Wanninger: andreas.wanninger@univie.ac.at

Orcid ID: 0000-0002-3266-5838

Christiane Todt: christiane.todt@bm.uib.no

*Corresponding author: Tim Wollesen; Email: tim.wollesen@univie.ac.at

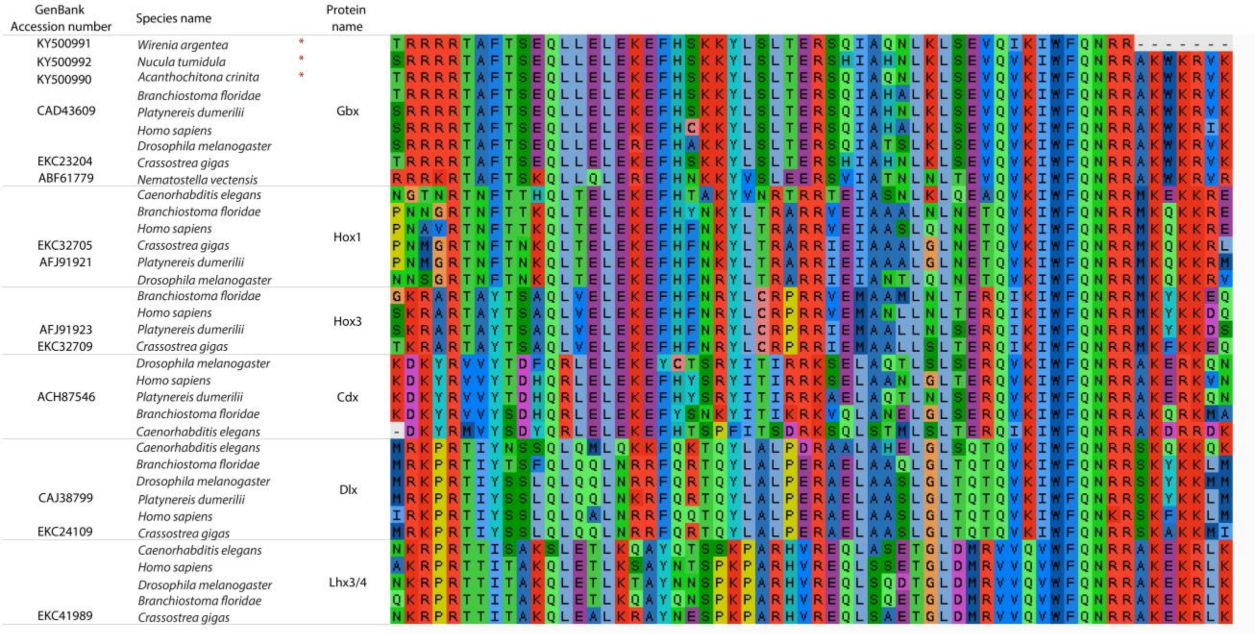

Supplementary Figure 1. Multiple amino acid sequence alignment of metazoan homeobox homeodomains including Gbx.

Homeodomain amino acid sequences of Gbx, Dlx, Hox1, Hox3, Cdx, and Lhx2/4 were retrieved from the homeobox database (http://homeodb.cbi.pku.edu.cn/) and GenBank (accession numbers provided in Figure)[21,22]. The deduced amino acid sequences of Acr-Gbx, War-Gbx, and Ntu-Gbx are highlighted with asterisks. The multiple sequence alignment was performed with MAFFT v7.123b[23], edited manually with Aliview v1.18, and used for the phylogenetic analysis shown in Supplementary Figure 2.

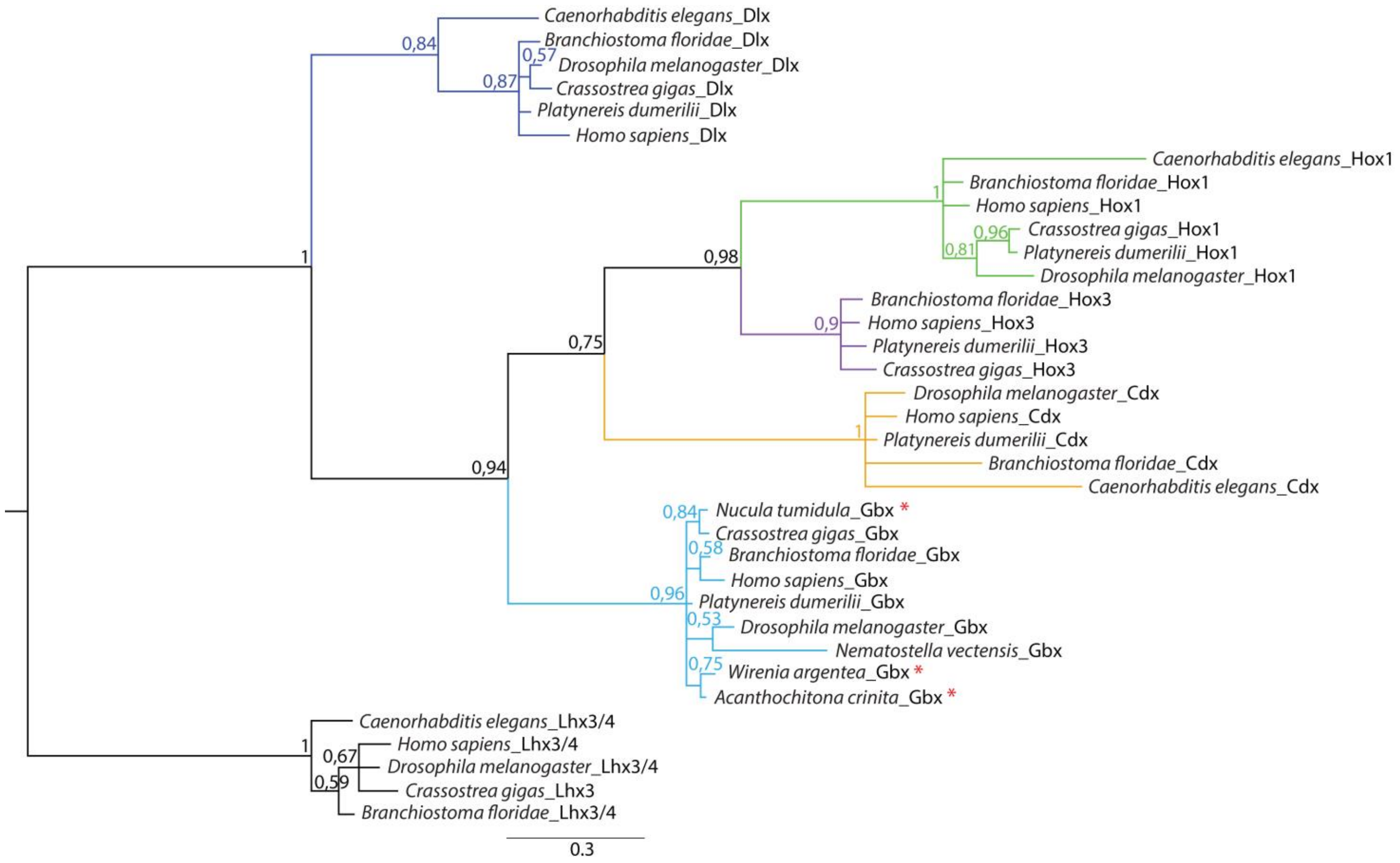

Supplementary Figure 2. Phylogenetic analysis of homeobox homeodomain amino acid sequences of selected metazoans including Gbx orthologs. Bayesian phylogenetic analysis was carried out with MrBayes v3.2.5 with LG model of amino acid replacement[24] estimated with Prottest3 v3.4.2. [25], gamma-distributed rates, 22.500.000 generations and sampling frequency of 1000. The phylogenetic tree was manually rooted using FigTree v1.4.1.[26].

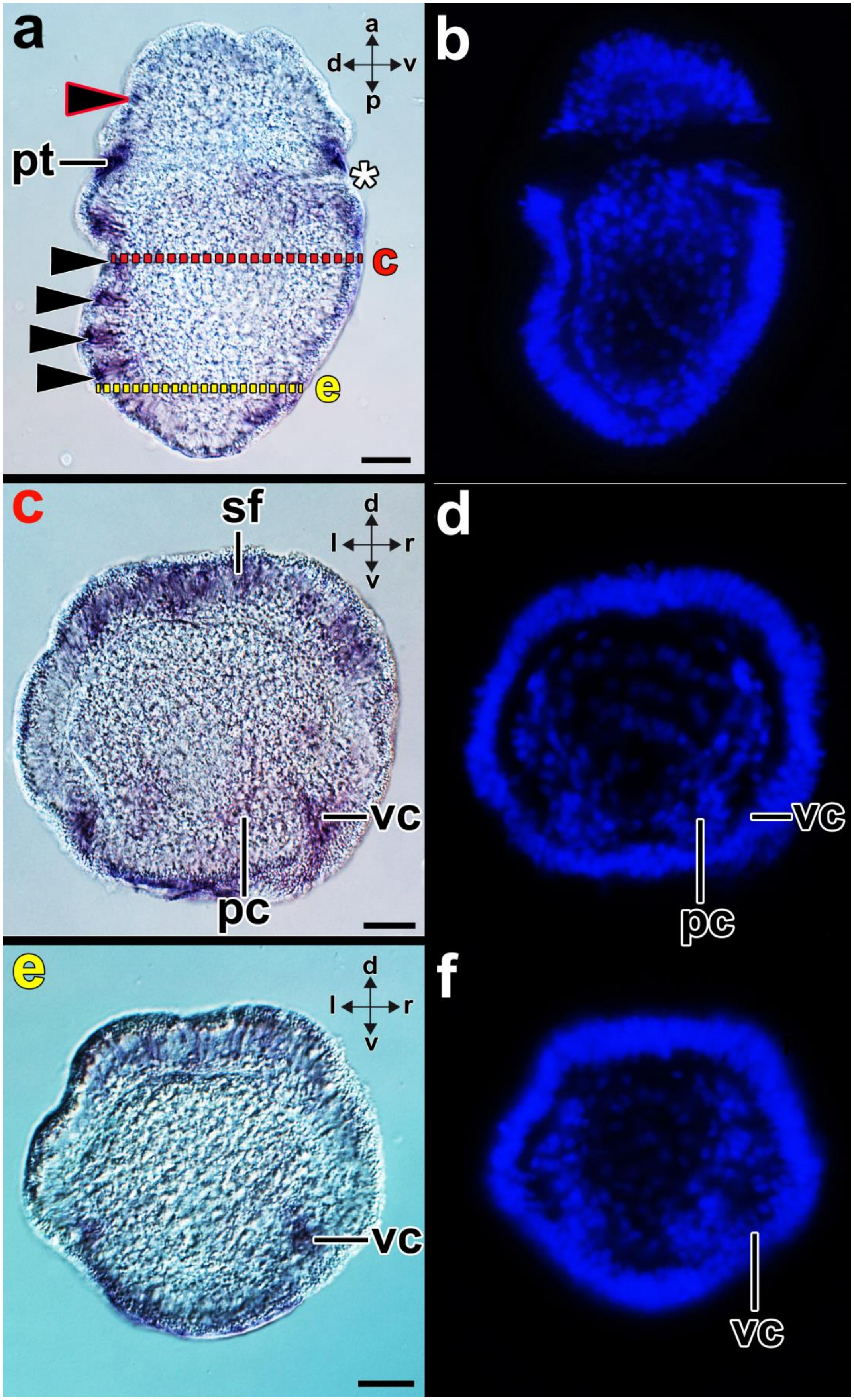
a
a
d
v
p
pt
c
e
b
c
sf
d
l
r
v
vc
pc
d
vc
pc
e
d
l
r
v
vc
f
vc

Supplementary Figure 3. Expression of *Gbx* in a mid-stage trochophore larva (35 hpf) of the polyplacophoran *Acanthochitona crinita.* Dorsal (d)-ventral (v), anterior (a)-posterior (p), and left (l)-right (r) axes indicate the orientation. All micrographs are cryostat sections with DIG-labeling (left column) and a nuclear stain (DAPI) (right column). **(a,b)** Sagittal section with *Gbx*-expression in the spicule-bearing cells (red-lined arrowhead) of the episphere and cells of the shell fields (arrowheads). The asterisk labels the mouth and the prototroch (pt) exhibits unspecific staining. **(c,d)** This transversal section highlights *Gbx*-expression in the visceral (vc) and pedal nerve cords (pc) and the shell fields (sf) of the anterior hyposphere. **(e,f)** Transversal section through a more posterior region of the hyposphere with *Gbx*-expression in the visceral nerve cords. Scale bars: 20 µm.

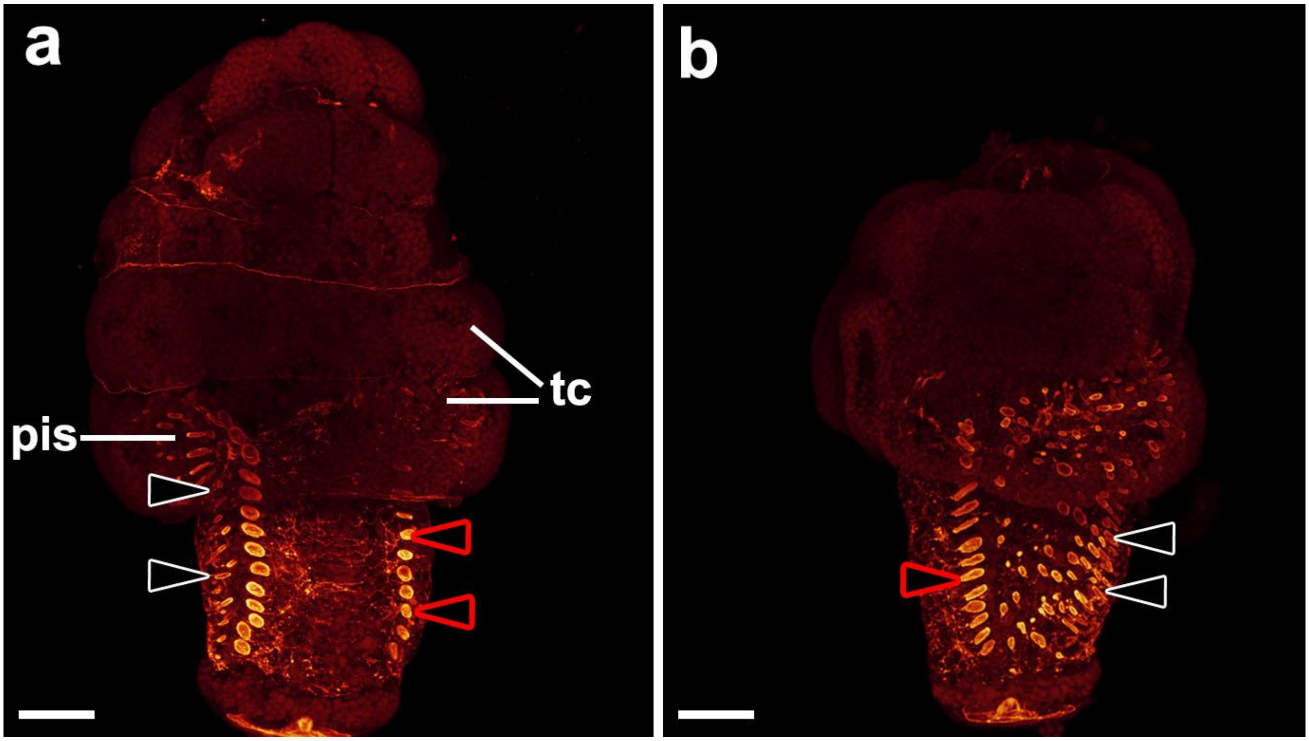

Supplementary Figure 4. Spicule-bearing cells in early test-cell larvae (7 dph) of the solenogaster *Wirenia argentea* visualized by fluorescence labeling of F-actin with Alexa Fluor 488 phalloidin. Anterior faces up. F-actin stained with phalloidin. **(a)** The developing spicule-bearing cells located along the prospective foot (red-lined arrowheads) on the ventral side of the outgrowing trunk are larger compared to those covering the remaining mantle (white-lined arrowheads) (ventral view). Note the peri-imaginal space (pis) and the large test-cells. **(b)** Lateral view of the same individual seen in A (ventral faces to the left). Scale bars: 20 µm.
